# Social support and self-efficacy during early adolescence: Dual impact of protective and promotive links to mental health and wellbeing

**DOI:** 10.1371/journal.pgph.0003904

**Published:** 2024-12-31

**Authors:** Megan Cherewick, Rinzi Lama, Roshan P. Rai, Choden Dukpa, Dikcha Mukhia, Priscilla Giri, Michael Matergia

**Affiliations:** 1 Department of Community & Behavioral Health, Colorado School of Public Health, University of Colorado Anschutz Medical Campus, Aurora, Colorado, United States of America; 2 Darjeeling Ladenla Road Prerna, Darjeeling, West Bengal, India; 3 Department of Anthropology, University of North Bengal, Darjeeling, West Bengal, India; 4 Broadleaf Health & Education Alliance, Stroudsburg, PA, United States of America; 5 Center for Global Health, Colorado School of Public Health, Aurora, Colorado, United States of America; PLOS: Public Library of Science, UNITED STATES OF AMERICA

## Abstract

This study aimed to assess the impact of sources of social support and dimensions of self-efficacy on psychological symptoms and mental wellbeing among early adolescents. A total of 274 adolescents aged 10–14 from Darjeeling, India, participated in the study. The Multidimensional Scale of Perceived Social Support (MSPSS) and Self-Efficacy Questionnaire for Children (SEQ-C) were utilized to assess dimensions of protective/promotive factors. Nested multivariable regression models assessed associations between age, gender, social support, and self-efficacy on psychological symptoms (internalising, externalising, and total difficulties) and mental wellbeing outcomes (mental wellbeing, optimism, and resilience). Results indicated that 13% of early adolescents screened positive for clinical depression and 44% reported poor mental wellbeing. Emotional and academic self-efficacy, along with family support, were significantly associated with reduced psychological symptoms. Conversely, social, and academic self-efficacy, along with support from friends, were linked to higher levels of mental wellbeing. Regression analyses revealed that dimensions of social support and self-efficacy explained a greater proportion of variability in mental wellbeing outcomes (R^2^ = 0.37–0.64) than in psychological symptom outcomes (R^2^ = 0.19–0.22), suggesting a stronger promotive effect on mental wellbeing compared to a protective effect on psychological symptoms during early adolescence. Findings suggest the urgent need for early mental health intervention to strengthen systems of social support and support self-efficacy among early adolescents. Multi-level or sequential interventions that target protective and promotive factors are a key strategy to addressing the global youth mental health crisis.

## Introduction

The Lancet Commission has underscored the rapid growth of adolescent in low and middle-income countries (LMIC) and the urgent need to identify protective and promotive factors for mental health during adolescence [1–3]. Early adolescence, typically defined as spanning ages 10–14, is increasingly recognized as a pivotal developmental period, marked by the onset of puberty. This stage offers a unique opportunity for targeted prevention interventions to enhance key protective and promotive factors for mental health and wellbeing, with potential long-lasting effects into mid to late adolescence, a time when the risk for mental health disorders often increases [3–9]. A recent meta-analysis of 192 epidemiological studies found that 34.6% of mental disorders have an onset before the age of 14 [10]. The proportion of disorders with onset prior to age 14 include neurodevelopmental disorders (61.5%), anxiety/fear-related disorders (38.1%), obsessive-compulsive/related disorders (24.6%), feeding/eating disorders (15.8%), conditions associated with stress disorders (16.9%), substance use disorders/addictive behaviors (2.9%), schizophrenia-spectrum disorders (3%), personality disorders/related traits (1.9%), and mood disorders (2.5%) [10]. Among the modifiable factors during early adolescence, social support and self-efficacy stand out as promising targets for prevention programs [11–16].

Understanding the connections between sources of social support (e.g., family, friends, significant others) and dimensions of self-efficacy (e.g., emotional, social, academic) with mental health and wellbeing outcomes is crucial. Social support involves the exchange of resources, whether tangible (e.g., financial) or emotional, between individuals [17–22]. Research consistently shows that social support is a critical protective factor for mental health, while its absence is often associated with poorer mental health outcomes [23–29]. The effectiveness of social support is influenced by individual, intrapersonal, and community-level factors, including age, gender, and socioeconomic status [30].

Cultural and contextual factors can also shape how social support impacts mental health during adolescence. For example, in collectivist societies that value close interpersonal relationships and communal resource-sharing, social support may have stronger protective and promotive effects on mental health and wellbeing [31, 32]. Studies have shown that social support significantly enhances life satisfaction, self-esteem, and overall mental health outcomes. For instance, research on Jordanian adolescents found that social support protected against mental health problems and improved life satisfaction and self-esteem [33]. Similarly, a study on Nepalese adolescents revealed that social support indirectly boosts psychological wellbeing through the mediating role of self-esteem [34]. Longitudinal research on Australian adolescents found that peer support can predict mental wellbeing, even in the absence of substantial family or significant other support [35]. Instruments like the Multidimensional Scale of Perceived Social Support (MSPSS) have been developed to capture these multifaceted aspects of social support [36]. To fully understand the impact of social support, it is important to explore how different types of support may have protective or promotive effects during different stages of development.

Self-efficacy is another key modifiable factor for mental health and wellbeing. Defined as the belief in one’s ability to execute a desired action, complete a novel task, or cope with a wide range of stressors, self-efficacy plays a crucial role in how individuals assess stressors and is closely linked to mental health and psychological wellbeing [37–42]. The development of self-efficacy during adolescence is shaped by dynamic interplay between individual capacities and environmental factors, which in turn influences behavior patterns in specific contexts. Studies have shown that higher self-efficacy is associated with lower levels of internalizing symptoms, such as depression and anxiety [37, 38, 43–46], and with positive mental health indicators like optimism and life satisfaction [38, 47]. While the role of self-efficacy in mitigating stress is well-documented, further research is needed to understand its contribution to promoting mental wellbeing [48]. The Self-Efficacy Questionnaire for Children (SEQ-C) is one tool used to assess the social, emotional, and academic dimensions of self-efficacy [49].

An estimated 90% of the global adolescent population live in LMIC, yet less than 10% of mental health trials have been conducted in these settings, and less than 1% with children and adolescents [1–3, 50]. With one-fifth of the global adolescent population, and only 1.9 mental health workers per 100,000 people (compared to 71.7 per 100,000 in high-income countries). In India, 33% of youth are affected by mental health disorders, including mood disorders such as depression and anxiety and behavioral disorders such as conduct disorder and substance misuse [3, 51–54]. India is a diverse country. The Darjeeling Himalaya is a remote mountainous region of India in the state of West Bengal. Darjeeling has a contested history of formation with a colonial history, as well as continual manifestations of regional autonomy demands. Programs to address the youth mental health crisis within India must consider the unique socio-ecological contexts that exist and to identify promotive and protective factors linked to mental health within specific contexts in India [52].

### Study objectives

This study aims to evaluate associations between age, gender and protective and promotive modifiable factors associated with psychological symptoms and mental wellbeing among early adolescents from in Darjeeling, India. By comprehensively examining these modifiable factors, the research seeks to illuminate the protective and promotive associations that can inform targeted intervention strategies during this critical developmental phase.

## Materials and methods

### Setting

This study was conducted in four schools representing urban, peri-urban, and rural areas of the Darjeeling Himalayas, a district in West Bengal, India. Darjeeling is known for its unique mountain geography and diverse ethnic composition, primarily consisting of Indian citizens of Nepali descent, along with other ethnic groups and economic migrants from nearby regions. The socioeconomic landscape is diverse but largely characterized by poverty, with most adults earning less than $2.76 USD per day [55]. The local economy is mainly driven by tea production, tourism, small-scale agriculture, and military service. Informal work and trade are common, and seasonal migration is a frequent practice among residents. In Darjeeling, low-cost private schools serve approximately 30–50% of all youth and youth increasingly attend these schools [55]. Each school enrolls approximately 200 students and receive minimal governmental support.

### Sample size and power

Sample size and power analyses for this study were conducted using G*Power 3.1.9.4. We conducted an a priori power analysis with α = 0.05, Power (1-β) = 0.95, and a two-tailed test for linear multiple regression. Our study was powered to detect an effect size with eight independent variables. The analysis indicated that a minimum sample size of N = 262 would be required to detect a small but meaningful effect size of β = 0.05 with 95% power, as has been established in prior studies [56, 57].

### Participants

The study involved four schools selected based on positive relationships with a local non-governmental organization and the implementation team. The study team first held meetings with school principals to explain the purpose of the study. School principals signed a Memorandum of Understanding detailing the potential risks and benefits of participating in the study. Classrooms with eligible adolescents were identified in collaboration with school leaders, and classrooms were then randomly selected to participate in the study. Eligibility criteria were: 1) ages 10–14, 2) resident of Darjeeling, India at the time of the study, 3) attending 1 of 4 low-cost private schools, 4) caregiver provided written consent, and 5) participant provided verbal assent. All eligible students within the selected classrooms were invited to participate. Participants and their caregivers were given a description study, that their participation was voluntary, and that they were allowed to withdraw from the study at any time. Informed written consent was obtained from caregivers of all adolescents, defined as the primary guardian of adolescents at the time of the study. All consent forms were read aloud the consent form in the local language, Nepali. After obtaining informed consent from caregivers of eligible adolescents, the study team scheduled data collection with adolescents and their caregivers. Informed assent from adolescents was obtained at the time of data collection. For this study, our goal was to best capture responses for all youth ages 10–14 attending school in Darjeeling and therefore there were no exclusion criteria. A trained member of the study team verbally read the assent script to adolescents in quiet, private space on school grounds. Included in the assent script, was a description of the study, that participation was voluntary, and that they may withdraw from the study at any time. Adolescents had the opportunity to ask questions and provide verbal indication of their assent to participation.

All research assistants collecting consent/assent from participants were trained in human subjects research and responsible conduct of research. Survey data was collected from September 8^th^, 2023-October 3^rd^, 2023. Surveys were translated from English to Nepali and back translated by two professional translators. Surveys were pilot tested with a small sample of adolescents (N = 10) and reviewed by the study team to ensure interpretability. Surveys were administered in 45-minute sessions after school, in a private location on school grounds. Research assistants read each question aloud in Nepali while participants recorded their responses on a paper survey available in Nepali or English (based on preference). To minimize bias, study team held meetings with caregivers and youth to clearly detail the study, potential risks and benefits, voluntary participation and confidentiality protocols, and importantly that participation in the study would in not affect academic grades. All participants and their caregivers were provided with referral/contact information for local mental health professionals community social workers, and the contact information for the local study team. After the survey, responses were entered into the secure Research Electronic Data Capture (REDCap) application by the research assistants. De-identification of data was completed to support participant confidentiality. The de-identified data is available in S1 Data section of this article. The study team follow rigorous data management and security protocols, maintained detailed records of the research process and held weekly or biweekly reflective discussions to reflect findings. All research participants received a small participant incentive (equivalent to $2USD), determined to be culturally appropriate in this context, and to compensate for time and transportation.

### Ethical approval

This study was approved by the Colorado Multiple Institutional Review Board (COMIRB) Protocol Number: 23–1421.

### Measures

#### Multidimensional Scale of Perceived Social Support (MSPSS)

The MSPSS self-report scale was designed to assess three dimensions of social support through twelve items [36]. The scale includes three subscales: family support (items 3, 4, 8, and 11), friend support (items 6, 7, 9, 12), and significant other support (items 1, 2, 5, 10). Responses are given on a 7-point Likert scale, ranging from 1 (strongly disagree) to 7 (strongly agree), allowing for greater response variability. Example items include: “*I have friends with whom I can share my joys and sorrows*”, and “*I can talk about my problems with my friends*” (friend subscale), “*My family really tries to help me*”, and “*I get the emotional help and support I need from my family*” (family subscale), and “*There is a special person in my life who cares about my feelings*”, and “*There is a special person who is around when I am need*” (significant others subscale). The total MSPSS score ranges from 12 to 84, with higher scores indicating greater perceived social support. The 3-factor structure of the MSPSS has been widely validated, with good internal consistency and test-retest reliability [36]. In this sample, the MSPSS showed excellent reliability with a Cronbach’s alpha of 0.85.

#### Social Emotional Efficacy Questionnaire for Children (SEQ-C)

The SEQ-C is a 24-item questionnaire developed by Muris et.al, (2001) to assess self-efficacy in children and adolescents across three domains: academic, social, and emotional. Each subscale contains eight items. Example items include: “*I can study for a test*”, and “*I can finish my homework every day*” (academic self-efficacy), “*I can become friends with other children*” and “*I can tell a friend I don’t feel well*” (social self-efficacy), “*I can control my feelings*” and “*I can succeed in being calm even when I’m scared*” (emotional self-efficacy). Responses are given on a 5-point Likert scale ranging from 1 (strongly disagree) to 5 (strongly agree), with higher scores indicating higher self-efficacy. The social self-efficacy subscale was used to assess concurrent validity in this sample, with the SEQ-C demonstrating good reliability (Cronbach’s alpha = 0.82).

#### Strengths and Difficulties Questionnaire (SDQ)

The SDQ is a 25-item questionnaire to assess mental health problems in children and youth aged 4–17 [58]. It includes five subscales: conduct problems, hyperactivity/inattention, emotional symptoms, peer problems and prosocial behavior. Broader internalizing (emotional symptoms and peer problems) and externalizing (conduct problems and hyperactivity-inattention) subscales can be calculated for population screening [59]. Total difficulties are the sum of all subscales except for prosocial behavior, with scores ranging from 0 to 40. Higher scores indicate greater mental health difficulties. Responses are recorded on a 3-point scale (0 = not true, 1 = somewhat true, 2 = certainly true). The SDQ showed acceptable reliability in this sample with a Cronbach’s alpha of 0.73.

#### The Child and Youth Resilience Measure-Revised (CYRM-R)

The CYRM-R is a 17-item self-report measure that assesses resilience across two subscales: intra/interpersonal resilience and caregiver resilience [60]. The measure is widely used in diverse cultural contexts and translated into over 20 languages [61]. Responses are recorded on a 5-point Likert scale ranging from 1 (strongly disagree) to 5 (strongly agree), with higher scores indicating greater resilience. The CYRM-R demonstrated good reliability in this sample with a Cronbach’s alpha of 0.82.

#### The WHO-5 mental well being index (WHO-5)

The WHO-5 is a short scale that measures subjective wellbeing through five items [62]. Responses are recorded on a 6-point Likert scale ranging from 0 (at no time) to 5 (all the time). The raw score (0–25) is multiplied by 4 to yield a final score ranging from 0 to 100, with higher scores indicating better mental wellbeing. Scores below 28 indicate a positive screen for clinical depression, and scores below 40 suggest poor mental wellbeing. Example items include “*I have felt cheerful and in good spirits*” and “*I have felt calm and relaxed*”. The WHO-5 had a Cronbach’s alpha of 0.71 in this sample.

#### The Life Orientation Test-Revised (LOT-R)

The LOT-R is a 10-item self-report measure of optimism, defined as a general expectation that good things will happen [63]. The scale includes two subscales: optimism (7 items) and pessimism (3 items). Example items include: “*In uncertain times I expect the best*” (optimism) and “*I expect more bad things to happen to me than good”* (pessimism). Responses are given on a 5-point Likert scale from 0 (strongly disagree) to 4 (strongly agree). Pessimism items are reverse coded to calculate a total optimism score, with higher scores indicating greater optimism. The LOT-R reliability in this sample was 0.57.

#### Additional measures

Additional demographic variables include sex, age, school, and class level.

#### Data analysis

Data analysis was completed using Stata Version 14. Results accounted for clustering at the school level to adjust for correlation of unmeasured variables within communities and unintended bias in sample selection. Multivariate regression analysis was conducted to establish the association between social support, self-efficacy and mental health and wellbeing outcomes. Regression analyses assessed dimensions of social support (friends, family, and significant others) and self-efficacy (academic, social, and emotional self-efficacy). All analyses were adjusted for age and gender because adolescent internalizing and externalizing symptoms tend to increase with age and show different trajectories by gender. Model building proceeded by generating a baseline model that included main covariates of age and gender. Next, in Model 2, sources of social support were included. Finally, in Model 3, dimensions of self-efficacy were included. All models report the percent of variation explained by each model step and the associated p-value for that model.

## Results

### Descriptive statistics

The analytical sample included 135 female and 139 male participants for a total of 274 participants ages 10–14 (Table 1). The mean age in the study sample was 12.4 (SD: 1.3). ANOVA tests were completed to assess for differences by gender in age, class, and location and indicated there were no significant differences by gender. Most participants lived in rural areas N = 198 (72.3%), followed by peri-urban locations N = 57 (20.8%), and urban locations N = 19 (6.9%).

**Table 1:** Descriptive statistics of the analytical sample (N = 274).

Sex	N (%)
Female	135 (59.3)
Male	139 (50.7)
Age	
10	24 (8.8)
11	55 (20.1)
12	59 (21.5)
13	60 (21.9)
14	76 (27.7)
Mean (SD)	12.4 (1.3)
Class	
5	41 (15.0)
6	56 (20.5)
7	52 (19.1)
8	66 (24.2)
9	44 (16.1)
10	14 (5.3)
Location	
Urban	19 (6.9)
Peri-urban	57 (20.8)
Rural	198 (72.3)

Table 2 lists means, standard deviations, and t-tests statistics to evaluate differences in measured variables by sex. There were no significant differences in MSPSS dimensions or total MSPSS score by sex. Emotional self-efficacy (t = -2.42; p = 0.016), social self-efficacy (t = -3.15; p = 0.002), and total self-efficacy (t = -2.24; p = 0.026) was higher in males than females. Total internalizing symptoms were higher in females than males (t = 5.10; p< = 0.001). Total difficulties as measured by the SDQ was higher in females than males (t = 3.88; p = < = 0.001).

**Table 2:** Descriptive statistics of measured variables compared by sex.

	Female		Male		Total		t-test	
	Mean	SD	Mean	SD	Mean	SD	t	p
MSPSS								
Friends	22.0	0.44	21.4	0.46	21.7	0.32	0.99	0.325
Family	23.8	0.41	23.5	0.37	23.7	0.28	0.47	0.637
Significant Others	22.1	0.43	21.7	0.49	21.9	0.33	0.58	0.565
Total MSPSS	67.9	0.99	66.6	1.04	67.3	0.72	0.93	0.354
SEQ-C								
Emotional	29.1	0.49	30.7	0.42	29.9	0.32	-2.42	0.016*
Social	30.3	0.40	32.1	0.41	31.2	0.29	-3.15	0.002**
Academic	30.9	0.41	30.9	0.44	30.9	0.30	0.06	0.956
Total SEQ	90.3	1.05	93.6	1.04	92.0	0.75	-2.24	0.026*
SDQ								
Internalizing symptoms	8.0	0.31	5.9	0.28	6.9	0.22	5.10	0.000***
Externalizing symptoms	7.0	0.26	6.6	0.29	6.8	0.19	1.09	0.277
Total difficulties	15.1	0.48	12.5	9.46	13.7	0.34	3.88	0.000***
WHO-5								
Depression	0.16	0.37	0.10	0.30	0.13	0.34	1.53	0.128
Poor Wellbeing	0.43	0.50	0.40	0.49	0.42	0.49	0.69	0.489
Mental Wellbeing Total	52.1	1.84	57.2	1.93	54.7	1.34	-1.93	0.055
LOT-R								
Optimism	25.3	0.44	26.9	0.45	26.1	0.32	-2.66	0.008**
CYRM								
Resilience	71.4	0.65	71.5	0.75	71.5	0.50	-0.10	0.920

Note.

*p<0.05

**p<0.01

***p<0.001

There were no significant differences by gender in total mental wellbeing scores. Following the WHO-5 wellbeing index conventional screening classifications, total scores less than 28 indicate a positive screen for clinical depression. In the sample, N = 36 (13.1%) of all early adolescents screened positive for depression. There were no significant differences in depression screening by sex. The WHO-5 also classifies those with scores under 50 as having “poor mental wellbeing”. In the sample, 114 (41.6%) of adolescents had scores under 50, or poor mental wellbeing. There was no significant difference in poor mental wellbeing by sex. As measured by the LOT-R, males had higher scores for optimism in comparison to females (t = -2.66; p = 0.008). There was no significant difference in total resilience scores by sex.

Table 3 presents a correlation matrix of all measured variables. Several variables included in this study were significantly correlated. Sex was positively correlated with emotional self-efficacy (r = 0.15; p = 0.016) and optimism (r = 0.16; p = 0.008); and negatively correlated with internalizing symptoms (r = -0.30; p< = 0.001) and total difficulties (r = -0.23; p< = 0.001). Age was negatively correlated with the family support subscale of the MSPSS (r = -0.24; p< = 0.001); the significant others subscale (r = -0.18; p< = 0.001); social self-efficacy subscale (r = -0.15; p = 0.013), the academic self-efficacy subscale (r = -0.24; p< = 0.001) and resilience (r = -0.24; p< = 0.001). The friends and family subscales of the MSPSS were significantly and positively associated with the significant other subscale of the MSPSS, mental wellbeing, optimism, and resilience; and negatively associated with internalizing and externalizing symptom subscales and total difficulties.

**Table 3:** Spearman correlation coefficients for key analytic variables.

Key Variables	1	2	3	4	5	6	7	8	9	10	11	12	13	14
1. Sex	-													
2. Age	-0.05	-												
3. Friends MSPSS	-0.06	0.04	-											
4. Family MSPSS	-0.08	-0.26***	0.32***	-										
5. Significant Others MSPSS	-0.01	-0.22***	0.52***	0.34***	-									
6. Emotional Self Efficacy	0.13*	-0.10	0.33***	0.34***	0.34***	-								
7. Social Self Efficacy	0.19**	-0.16*	0.40***	0.32***	0.39***	0.59***	-							
8. Academic Self Efficacy	-0.01	-0.26***	0.29***	0.45***	0.32***	0.49***	0.45***	-						
9. Internalising Symptoms	-0.30***	0.01	-0.15**	-0.15***	-0.12*	-0.27***	-0.25***	-0.13*	-					
10. Externalising Symptoms	-0.08	0.16**	-0.09*	-0.23***	-0.05	-0.28***	-0.19**	-0.43***	0.36***	-				
11. Total Difficulties	-0.23***	0.11	-0.15**	-0.24***	-0.12*	-0.34***	-0.29***	-0.35***	0.83***	0.80***	-			
12. Mental wellbeing	0.10	-0.10	0.39***	0.35***	0.38***	0.45***	0.49***	0.44***	-0.28***	-0.29***	-0.35***	-		
13. Optimism	0.16**	-0.11	0.42***	0.34***	0.32***	0.46***	0.49***	0.45***	-0.23***	-0.23***	-0.27***	0.38***	-	
14. Resilience	0.02	-0.27***	0.55***	0.58***	0.47***	0.54***	0.60***	0.52***	-0.22***	-0.25***	-0.28***	0.56***	0.51***	-

Note.

*p<0.05

**p<0.01

***p<0.001

Similarly, the significant others subscale of the MSPSS was significantly associated with the same variables as the friends and family subscales except for externalizing symptoms, where no significance was observed. Emotional, social, and academic self-efficacy subscales were positively and significantly associated with mental wellbeing, optimism and resilience and negatively associated with internalizing and externalizing symptoms and total difficulties. Outcome variables of mental health (internalizing, externalizing and total difficulties) and mental wellbeing outcomes (mental wellbeing, optimism, and resilience) were inversely and significantly associated.

Results of multivariable regressions of measured variables on psychological symptoms are presented in Table 4. In Model 1 (M1), sex and age covariates were included in the baseline regression model. In Model 2 (M2), subdimensions of the MSPSS (friends, family, and significant others) were included. In Model 3 (M3), subdimensions of the SEQ-C (academic, social, and emotional self-efficacy) were included for each measured outcome of psychological symptoms. In M1, males had significantly lower internalizing symptoms (β = -2.12; p< = 0.001). In M2, the significant negative association between sex and internalizing remained (β = -2.25; p< = 0.001). Family support was negatively associated with internalizing symptoms (β = -0.15; p = 0.003). In M3, the significant relationship between sex and internalizing attenuated (β = -1.92; p< = 0.001), as did family support (β = -0.12; p = 0.028), though both remained significant. Emotional self-efficacy was associated with lower internalizing symptoms (β = -0.12; p = 0.013). M3 accounted for 19% of the variation in internalizing symptoms (p< = 0.001).

**Table 4:** Nested multivariable regressions on psychological symptoms.

		Internalising					Externalising					Total Difficulties				
Model		β	SE	p	R^2^	Mp	β	SE	p	R^2^	Mp	β	SE	p	R^2^	Mp	
*M1*					0.09	0.000***				0.03	0.019*				0.06	0.000***	
	Age	-0.00	0.15	0.991			0.38	0.15	0.009**			0.38	0.25	0.139			
	Sex	-2.12	0.42	0.000****			-0.35	0.38	0.354			-2.49	0.67	0.000***			
*M2*					0.16	0.000***				0.09	0.000***				0.16	0.000***	
	Age	-0.15	0.16	0.368			0.25	0.15	0.085			0.10	0.25	0.683			
	Sex	-2.26	0.41	0.000***			-0.44	0.37	0.239			-2.70	0.64	0.000***			
	Friends	-0.09	0.05	0.065			-0.05	0.04	0.264			-0.14	0.07	0.064			
	Family	-0.15	0.05	0.003**			-0.16	0.04	0.000***			-0.31	0.08	0.000***			
	Significant Others	-0.02	0.05	0.685			0.02	0.04	0.699			0.00	0.07	0.967			
*M3*					0.19	0.010*				0.23	0.000***				0.22	0.000***	
	Age	-0.13	0.16	0.444			0.15	0.14	0.266			0.03	0.25	0.919			
	Sex	-1.92	0.42	0.000***			-0.38	0.36	0.282			-2.32	0.63	0.000***			
	Friends	-0.06	0.05	0.213			-0.02	0.04	0.619			-0.08	0.07	0.269			
	Family	-0.12	0.05	0.028*			-0.05	0.04	0.281			-0.16	0.08	0.040*			
	Significant Others	0.01	0.05	0.909			0.05	0.04	0.215			0.06	0.07	0.444			
	Emotional Self-Efficacy	-0.12	0.05	0.013*			-0.08	0.04	0.050*			-0.21	0.08	0.006**			
	Social Self-Efficacy	-0.06	0.06	0.298			0.06	0.05	0.204			0.00	0.09	0.967			
	Academic Self-Efficacy	0.05	0.05	0.288			-0.25	0.04	0.000***			-0.20	0.08	0.010*			

Note. SE = Robust standard errors

p*<0.05

**p<0.01

***p<0.001; R^2^ reported for each model step; Mp = p-value for the model step

In M1, for externalizing symptoms, age was positively and significantly associated with symptoms (β = 0.38; p = 0.009), however in M2 this association did not remain. In M2, family support was negatively and significantly associated with externalizing symptoms (β = -0.16; p<0.001). In M3, this association was no longer significant. Emotional self-efficacy (β = -0.08; p = 0.050) and academic self-efficacy (β = -0.25; p< = 0.001) were significantly associated with externalizing symptoms. M3 accounted for 23% of the variance in externalizing symptoms (p< = 0.001).

In M1 for total difficulties, male sex was associated with lower total difficulties (β = -2.49; p< = 0.001). This association remained significant in M2 (β-2.70; p< = 0.001). In M2 family support was negatively associated with total difficulties (β = -0.31; p< = 0.001). In M3, sex remained significantly associated with total difficulties after adjusting for all variables (β = -2.32; p< = 0.001). Family support remained significant (β = -0.16; p = 0.040). Emotional self-efficacy (β = -0.21; p = 0.006) and academic self-efficacy (β = -0.20; p = 0.010) were associated with lower levels of total difficulties. M3 accounted for 22% of the variability in total difficulties (p< = 0.001).

Results of multivariable regressions of measured variables on mental wellbeing outcomes are presented in Table 5. Mental wellbeing was not significantly associated with age or sex in M1; however, sex was positively and significantly associated with mental wellbeing in M2 (β = 6.09; p = 0.009). In M2, support from friends (β = 0.89; p = 0.001), family (β = 1.17; p< = 0.001) and significant others (β = 0.79; p = 0.004) were positively associated with mental wellbeing. In M3, adjusted for dimensions of self-efficacy, support from friends remained significant (β = 0.53; p = 0.043), however sex, family support, and support from significant others were no longer significant. Social self-efficacy was significantly and positively associated with mental wellbeing (β = 1.08; p< = 0.001) as was academic self-efficacy (β = 0.91; p = 0.001). M3 accounted for 37% of the variance in mental wellbeing (p< = 0.001).

**Table 5:** Nested multivariable regressions on mental wellbeing and resilience outcomes.

		Mental Wellbeing					Optimism					Resilience				
Model		β	SE	p	R^2^	Mp	β	SE	p	R^2^	Mp	β	SE	p	R^2^	Mp	
*M1*					0.03	0.032*				0.03	0.011*				0.06	0.000***	
	Age	-1.90	1.01	0.061			-0.38	0.24	0.117			-1.53	0.37	0.000***			
	Sex	4.66	2.66	0.081			1.58	0.63	0.013*			-0.14	0.97	0.882			
*M2*					0.27	0.000***				0.21	0.000***				0.56	0.000***	
	Age	-0.25	0.92	0.783			-0.04	0.21	0.861			-0.57	0.27	0.036*			
	Sex	6.09	2.32	0.009**			1.95	0.53	0.000***			0.55	0.67	0.413			
	Friends	0.89	0.27	0.001**			0.33	0.06	0.000***			0.43	0.08	0.000***			
	Family	1.17	0.28	0.000***			0.30	0.06	0.000***			0.88	0.08	0.000***			
	Significant Others	0.79	0.27	0.004**			0.09	0.06	0.164			0.24	0.08	0.002***			
*M3*					0.37	0.000***				0.42	0.000***				0.64	0.000***	
	Age	0.38	0.87	0.666			0.09	0.20	0.658			-0.43	0.25	0.084			
	Sex	3.34	2.24	0.136			1.38	0.51	0.007**			-0.62	0.63	0.327			
	Friends	0.53	0.26	0.043*			0.25	0.06	0.000***			0.30	0.07	0.000***			
	Family	0.54	0.28	0.055			0.12	0.06	0.056			0.70	0.08	0.000***			
	Significant Others	0.40	0.26	0.128			0.00	0.06	0.981			0.11	0.07	0.131			
	Emotional Self-Efficacy	0.28	0.27	0.298			0.18	0.06	0.003**			0.18	0.08	0.019*			
	Social Self-Efficacy	1.08	0.31	0.000***			0.10	0.07	0.133			0.42	0.09	0.000***			
	Academic Self-Efficacy	0.91	0.27	0.001***			0.25	0.06	0.000***			0.13	0.08	0.105			

Note. SE = Robust standard errors

p*<0.05

**p<0.01

***p<0.001; R^2^ is reported for each model step; Mp = p-value for the model step

For the outcome optimism, male sex was positively associated with higher levels of optimism (β = 1.58; p = 0.013) in M1 and remained significant (β = 1.95; p< = 0.001) in M2. Support from friends (β = 0.33; p< = 0.001) and family (β = 0.30; p< = 0.001) were significantly associated with higher levels of optimism. In M3, sex remained significantly associated with optimism (β = 1.38; p = 0.007). Support from friends remained significant (β = 0.25; p<0.001). Emotional self-efficacy (β = 0.18; p = 0.003) and academic self-efficacy (β = 0.25; p< = 0.001) were significantly and positively associated with optimism. M3 accounted for 42% of the variation in optimism (p< = 0.001).

For the outcome resilience, each year increase in age was associated with decreased resilience (β = -1.53; p< = 0.001) in M1 and age remained significant though attenuated (β = -0.57; p = 0.036) in M2. Support from friends (β = 0.43; p<0.001), family (β = 0.88; p<0.001), and significant others (β = 0.24; p = 0.002) were associated with higher reported resilience. After adjusting for dimensions of MSPSS, age and sex, the support from family had the largest coefficient (β = 0.88) in relationship to resilience. In M3, only support from friends (β = 0.30; p< = 0.001) and family (β = 0.70; p< = 0.001) remained significant. Emotional self-efficacy (β = 0.18; p = 0.019) and social self-efficacy (β = 0.42; p< = 0.001) were positively associated with higher resilience. M3 accounted for 64% of the variation in resilience (p< = 0.001).

## Discussion

The study aimed to assess the relationship between dimensions of social support and self-efficacy, and their associations with mental health and wellbeing outcomes. In this sample of early adolescents from Darjeeling, India, male sex was linked to lower levels of internalizing symptoms and total difficulties. This finding aligns with other studies indicating that male sex can be protective for psychological symptoms during early adolescence [64–66]. The increase in internalizing symptoms typically seen with age during adolescence, combined with the fact that males generally experience puberty later than females, likely explains some of these observed effects. Cultural gender norms might also contribute; for instance, in the Democratic Republic of Congo, post-pubertal girls are often restricted from social interactions outside [67].

Family support in this study was linked to lower levels of internalizing symptoms and total difficulties. This is consistent with previous research, which has shown that a lack of perceived family support predicts depressive symptoms and suicidal ideation, while increased family support correlates with lower severity of depressive symptoms [25, 68]. Other types of social support, such as support from peers and significant others, also offer protection against internalizing symptoms. For example, in Ghana, perceived social support buffered the effects of academic stress on psychological wellbeing [69]. Additionally, during COVID-19, a study with Chinese adolescents found that social support mediated the impact on depression and anxiety symptoms [70]. Future research should explore the quality of social support within each subdimension. For instance, the presence of friends differs from the presence of high-quality friendships. Friendship quality in early adolescence could be a crucial factor in interventions aimed at enhancing peer support [71]. Moreover, while the MSPSS does not directly assess online social support, the growing engagement with social media during adolescence underscores the importance of investigating how social media can provide positive social support [72, 73].

Research suggests that self-efficacy mediates the relationship between stress and psychopathology [74]. In India, studies indicate that nearly two-thirds (63.5%) of students experience stress due to academic pressure, and 66% report stress related to caregiver relationships [75]. In this study, emotional self-efficacy was associated with lower levels of internalising symptoms. Prior research has found that academic and emotional self-efficacy are significantly negatively correlated with depressive symptoms [48, 49]. Additionally, higher levels of self-efficacy have been linked to lower externalizing behaviors [76]. Other studies have shown that academic self-efficacy is a better predictor of depressive symptoms than social and emotional self-efficacy [77]. In this study, both academic and emotional self-efficacy were associated with lower externalizing symptoms.

In this sample, social self-efficacy and peer support were associated with better mental wellbeing. The full model (M3), which included all measured variables, accounted for a greater proportion of variance in mental wellbeing outcomes compared to psychological symptoms. Specifically, M3 explained 37% of the variation in mental wellbeing, 42% in optimism, and 64% in resilience. These finding suggest that social support and self-efficacy may play a more significant role in promoting mental wellbeing than in protecting against psychological symptoms. Among the dimensions of self-efficacy measured, social self-efficacy had the strongest relationship with mental wellbeing. Longitudinal research is necessary to evaluate mechanistic pathways between mental wellbeing and psychological symptoms in middle and later adolescence changes during development. The relationship between mental wellbeing and psychological symptoms is likely to be complex and may include causal, reverse causal, or reciprocal relationships during specific period of development.

Interventions targeting early adolescence should consider addressing multiple dimensions of both social support and self-efficacy to improve mental health and wellbeing. An ideal approach would be to leverage synergistic effects of mental health promotion, prevention and treatment programs. In Darjeeling, a lay field-worker-led school health program for primary school age children improved health outcomes and health knowledge [78]. An indicated treatment trial, Teachers Leading the Frontline (Tealeaf), is an example of a promising, novel, task-shifted mental health care intervention for children ages 5–12, in Darjeeling, India [79]. A gap in these existing programs is the need for a targeted prevention program that modifies promotive and protective factors for mental health during early adolescence. Such a program seeking to modify social support, and self-efficacy may benefit from implementation programs delivered in peer groups. The heightened sensitivity to social reward, status, and admiration that characterizes early adolescence is a unique neurodevelopmental window to modify these protective and promotive factors [80]. Further, these protective and promotive factors may be critical for a diversity of youth, including youth with neurodevelopmental conditions, expanding the population reach and impact of prevention programs [81]. Research has identified key components of effective adolescent interventions [82]. Utilizing community based participatory research methods, and co-designing interventions with and for youth can amplify these key ingredients that include efforts to make programs authentic, agency-enhancing, and socially rewarding. Lastly, incorporating youth with lived experience in the design of these interventions can help support innovation and inclusivity in prevention programs, and better address the urgent need for effective and scalable prevention programs [83].

### Limitations

All measures were self-reported, and participant responses may have been influenced by social desirability bias. We attempted to minimize response bias by clarifying the importance of answering questions honestly, explaining that the study team had protocol measures in place to ensure confidentiality, and emphasizing that responses would in no way affect academic grades. Additionally, these quantitative findings were part of the larger Darjeeling Early Adolescent Study, which also included qualitative in-depth interviews and focus groups with youth ages 10–24, their caregivers, teachers, and community health workers to gain greater contextual depth of early adolescent mental health in this context. While qualitative results are not yet published, emergent themes were discussed by the study team from qualitative analyses were used to ground quantitative findings and interpretations presented in this study. These findings are specific to the context of Darjeeling, India, and may not be generalizable to other settings within our outside of India. Darjeeling is a remote, mountainous region with a unique geopolitical history. Convenience sampling of the four selected schools may limit generalizability. Future studies that randomly select schools based on location, school size, and socioeconomic factors will be important to support generalizability throughout the region. While inclusion criteria stated participants must be residents of Darjeeling, duration of residency prior to inclusion in the study was not specified. Given migration patterns that are common in this region, future studies may consider including measures of migration and duration of residency in Darjeeling. The age range of participants was limited to 10–14 years, and correlational associations between protective factors, mental health, and wellbeing outcomes are likely to change with development. The cross-sectional nature of the data prevents evaluation of causal directions, and future longitudinal studies are needed to examine how these associations evolve over time to identify causal directionality and critical windows for prevention programs. Finally, we believe that additional measures that capture family dynamics and contextual factors such as migration patterns and school climate would help to characterize the complexity of the relationships between social support, self-efficacy and mental health and wellbeing.

## Conclusion

The study’s results indicate that dimensions of social support and self-efficacy play significant roles in protecting against psychological symptoms and promoting mental wellbeing. Specifically, emotional self-efficacy and family support were more effective in protecting against psychological symptoms, while social self-efficacy and peer support were more influential in enhancing mental wellbeing. Given that social support and self-efficacy are modifiable factors, they may serve as mediators between stress and mental health outcomes. Mental health prevention programs targeting early adolescence should consider multi-level designs that engage peers, family, and community to address the global youth mental health crisis.

## Supporting information

S1 Text Inclusivity in global health. (DOCX)

S1 Data Deidentified dataset. (XLSX)
